# Pediatric neurosurgery AC-after COVID-19: What has really changed? A review of the literature

**DOI:** 10.3389/fped.2022.928276

**Published:** 2022-09-07

**Authors:** Alice Noris, Simone Peraio, Andrea Di Rita, Zaccaria Ricci, Chiara Spezzani, Matteo Lenge, Flavio Giordano

**Affiliations:** ^1^Neurosurgery and Functional Neurosurgery Unit, Department of Neurosurgery, Meyer Children's Hospital, University of Florence, Florence, Italy; ^2^Department of Anesthesiology and Intensive Care, Meyer Children's Hospital, University of Florence, Florence, Italy

**Keywords:** COVID-19, pediatric neurosurgery, hospital organization, surgical indications, perioperative complications, virtual meetings

## Abstract

The COVID-19 outbreak has dramatically changed the organization of Pediatric Neurosurgery all over the world. The departments involved developed similar plans to maintain emergency surgeries without reducing clinical activities. The Association of Pediatric Neurosurgeons wrote different memoranda to detail the surgical procedures not to be postponed with special attention given to high-risk pathology for COVID-19 contamination, like trans-naso-sphenoidal surgery. On this basis, we have conducted a complete literature review focusing on many topics: hospital organization, patients and parents screening, surgical indication criteria, outpatient clinic and teleconsultation, telematic conference and meeting, fellowship and training, and virtual multidisciplinary meeting.

## Introduction

Coronavirus disease (COVID-19) is an infectious respiratory disease that may cause a severe respiratory illness and is still threatening humanity globally. Since the first patients were infected in China in December 2019, it has been studied deeply by scientists from all the world. COVID-19 is caused by a new coronavirus of the Coronaviridae family (SARS-CoV-2). The virus structure is related to other viruses responsible for severe acute respiratory syndrome (SARS) (1). Due to COVID-19's long incubation period, high infection rate, and a variety of manifestations mainly affecting the respiratory function, but also with neurological symptoms in some cases, this infection has an unpredictable course and represents an unprecedented challenge to the modern health care system and society (2).

The rapid spread of the pandemic meant the global medical community faced both practical and ethical challenges that required an urgent response to adapt the way healthcare was provided. This was true for all the medical disciplines and also for the neurosurgery community (3). The literature has demonstrated that children affected by COVID develop a less severe form of infection compared to adults (4). However, fatal cases have already been described in some countries, such as the US, the UK, France, and Belgium. The mortality has been estimated around 0.08% among the pediatric population (5). Although COVID-19 in children may be milder, pediatric patients may need hospital medical support and continuous assistance. Furthermore, even if they are affected by COVID-19, they still require the same surgical and non-surgical assistance they needed before (6). Due to the consistent need for pediatric neurosurgical care (trauma, tumors, malformation, etc.) and the new request of COVID-19 treatment, the role of pediatric neurosurgical care in global public health has changed considerably. In fact, healthcare providers, hospitals, and, most importantly, neurosurgeons from all around the world have had to change their mind regarding their protocols. The clinical needs of patients affected by neurosurgical conditions now need to be balanced with the new restrictions and limitations that hospitals face due to the Covid pandemic. The aim of this study was to evaluate the level of evidence and new guidelines established in the world to face the new needs deriving from COVID-19 and neurosurgical pathologies.

## Results

We conducted a comprehensive literature search on Pubmed for publications including “COVID-19,” “Neurosurgery,” and “Pediatric population” as key words. Out of 18 papers, seven articles met the inclusion criteria. One of the main challenges faced in the article selection was related to the reliability of the supporting data, which were mostly obtained from reports that emerged early during the pandemic, and how the COVID-19 pandemic impacted on the pediatric neurosurgical population, which was less explored compared to the adult one. The inclusion parameters were based on the wide diversity of study methodologies, quality of publication, statistical approaches, sample sizes, population characteristics, and geographic and timing parameters. Therefore, 11 articles were excluded because of some limitations of the result's stratification, or the investigation's inaccuracy around surgical procedures and coding diagnosis. Some articles were not included because the results, as many studies related to perioperative complications, for example, were coming from pediatric patients with a general diagnosis of upper respiratory tract infections who did not specifically test positive for COVID-19. Others were excluded because they were investigated more on the anesthesiologic aspects and the pre/post-operative care rather than the actual neurosurgical intervention. The review of the seven articles selected showed different specific topics, such as the trend of neurosurgical operative volumes and solutions to manage the rate of underdiagnosis and the increased length of waiting lists (Table 1). Indications for neurosurgical procedures were analyzed, stratified by timing, and then divided into four main classes in order to have reliable decisional criteria. The studies on perioperative complications and incidence of anesthetic complications in COVID-19 positive children showed higher rates compared to the controls. Many multistep strategies in the pre-operative, anesthesia induction, extubation, and post-operative phases were hence developed to limit the increased risk of viral transmission.

**Table 1:** Summary of the most important topics affected by COVID-19 pandemic.

**Topic**	**Issue**	**Solution**	**Paper (Author)**
Decreased neurosurgical operative volumes (especially in the categories of epilepsy, spine, trauma, and shunt)	- Fear-related changes in health-seeking behavior and decreased consultation lead to underdiagnosis - Increased length of waiting list due to the postponement of elective surgeries and the increase in neurosurgical referrals	Clinical and academics institution updated their policy to permit in-person interactions and tried to decrease, together with vaccines, the rate of underdiagnosis which could precipitating even longer waiting lists for elective surgeries and the dependent volume of neurosurgical operations	Trends in United States Pediatric Neurosurgical Practice during the COVID-19 Pandemic (7)
Surgical indications	Decisional criteria in pediatric patients requiring urgent neurosurgical intervention and resulting positive to PCR test for SARS-CoV-2	Classification of neurosurgical procedures stratified by timing into 4 classes ranging from emergent and urgent procedures (class I) to neurosurgical conditions able to delay treatment more than 1–2 months (class IV)	Urgent Neurosurgical Interventions in the COVID-19-Positive Pediatric Population (8)
Perioperative complications and care	The incidence and severity of anesthetic complications in children with severe acute respiratory syndrome coronavirus 2 is unknown	1. Perianesthetic respiratory complications have higher rates in children with non -severe SARS-CoV-2 infection as compared to matched controls although severe morbidity was rare and there was no mortality 2. The incidence of complications for children with non-severe SARS-CoV-2 infection is similar to the one reported for an upper respiratory tract infection.	Anesthetic Complications Associated With Severe Acute Respiratory Syndrome Coronavirus 2 in Pediatric Patients (9)
	Increased risk of viral transmission to other patients and care providers due the high asymptomatic carrier rate in the pediatric population	1. Minimize crying in the pre-operation (premedication, non-pharmacologic anxiolysis…) 2. IV induction (consider RSI, minimize Bag Mask Ventilation) 3. Prevent coughing during extubation (deep extubation to with adjuncts) 4. Recover in Negative Pressure Isolation Room or in the Operating Room in the post-operation	Unique Challenges in Pediatric Anesthesia Created by COVID-19 (10)
Hospital organization	Policies and stratiegies to limit Covid-19 spread during surgical operations and patients stay at the hospital	1. Supply of adequate equipment 2. Proper training of the healthcare personnel 3. Effective protocols to minimize contact in the operating rooms 4. Patients and parents screening	Urgent Neurosurgical Interventions in the COVID-19-Positive Pediatric Population (8)
Outpatient clinic and patients follow up	Minimize traffic into the facility of patients and family members who may be COVID-19 positive and face-to-face interaction with staff and clinic personnel	Telemedicine and teleconsultation with improvements in training, billing, and credentialing for both telephone and video-based clinic visits	Editorial. Pediatric Neurosurgery along with Children's Hospitals' Innovations Are Rapid and Uniform in Response to the COVID-19 Pandemic (3)
Conference and multidisciplinary meeting	Decrease face-to-face interaction with staff and personnel to limit the potential spread of the virus	1.Daily morning report, educational and subspecialty conferences are conducted electronically by telephone or video conference 2. Daily telephone call or video conference led by service chiefs, to keep their entire teams updated on the rapidly evolving information on the pandemic	Editorial. Pediatric Neurosurgery along with Children's Hospitals' Innovations Are Rapid and Uniform in Response to the COVID-19 Pandemic (3)
Fellowship and training	Decrease face-to-face interaction with colleagues and personnel to limit the potential spread of the virus	More opportunities for “off-campus” work and telerotation	Editorial. Pediatric Neurosurgery along with Children's Hospitals' Innovations Are Rapid and Uniform in Response to the COVID-19 Pandemic (3)

The main issues, the related solutions, and the paper which described the topics are presented.

Regarding hospital organizations, the focus was on different polices and customized strategies to limit COVID-19 spread, such as protocols to minimize contacts, screening, adequate equipment, and update training of the personnel. Thanks to many improvements and innovations in telemedicine, teleconsultation, and other different online solutions, outpatients' clinics and follow ups, conferences, and multidisciplinary meetings do not need to be postponed. Finally, online meetings created a more “off-campus” experience to guarantee the adequate training and professional education of residents and fellows without increasing the number of contacts and face-to-face interactions.

## Discussion

The COVID-19 pandemic has considerably altered many aspects of neurosurgery. More papers have been published concerning adult settings, while pediatric reports are still few. We tried to review different topics including surgical indication criteria, hospital organization, perioperative complications and care, patients and parents screening, outpatient clinic and teleconsultation, telematic conference and meeting, virtual multidisciplinary meeting, and fellowship and training.

### Neurosurgical operative volume

Dave et al. described how numbers of neurosurgical operations lowered during the COVID-19 pandemic in 2020 and 2021 (7). The decrease in surgeries involved different subcategories but epilepsy, non-traumatic spine, and trauma were the most affected ones. They also recorded a decrease in shunt operations. The reduction in volumes could be due to different health-seeking behavior (people tried to avoid hospitals), underdiagnosis, and decrease in trauma cases related to the reduction in activities and quarantine.

Another factor is online education since absence seizures and changes in behavior indicative of shunt failure may be principally noticed in academic settings or during interactions with other people. Moreover, the shift of hospital resources toward medical specialties contributed to the postponement of some elective surgeries. In 2020 and 2021, the decrease in COVID-19 cases was accompanied by an increase in neurosurgical referrals and operations due to the long waiting lists for elective surgeries. However, institutions have now updated their policies and vaccines are becoming more widespread even among children, so the rebound of surgeries and referrals is less evident in 2022.

### Surgical indication criteria

Despite the overall reduction in surgical interventions, differently from other neurosurgical subspecialties, most pediatric neurosurgical cases are emergent or urgent because delayed operations can impede cognitive development. Therefore, institutions all over the world tried to adapt and develop protocols to guide healthcare personnel in their daily practice. Lang et al. conducted a retrospective study including pediatric patients who required urgent neurosurgical intervention and had a positive PCR test for COVID-19 (8). They stratified neurosurgical procedures based on the timing of the need for intervention and detailed all the challenges in dealing with COVID-19-positive patients who also needed urgent surgery. The procedures that could not be postponed included CSF diversion of hydrocephalus or hygromas, evacuation of hematomas/hemorrhages, stabilization of unstable fractures, and tumor resection. All these interventions could be safely performed without additional risks or worsening sequelae for the patients.

Lang et al. also described how the equipment should be adequate and the healthcare personnel must be properly trained to prevent the diffusion of the virus and to avoid complications for the patients (8). For example, premedication with midazolam before surgery has the potential benefit of minimizing crying or coughing by the infected child, thereby reducing the risk of aerosolization of COVID-19 viral particle. For the same purpose, complete muscle relaxation with intravenous neuromuscular blockade was utilized. To reduce air leakage, they used a cuffed orotracheal tube. No additional personnel were present at the induction of anesthesia to minimize the number of contacts and the waste of protective devices; the surgical team entered the room only after the patient was on mechanical ventilation. To minimize door opening, walkie-talkies or phones were used for communication with people outside the room. Movements from the rooms were limited as much as possible and visitors were not allowed. In the series by Lang and colleagues, no patient experienced worsening of COVID-19 related symptoms that required targeted therapy, apart from supportive care (8). They demonstrated that pre-, peri-, and post-operative care of COVID-19-positive patients requiring urgent neurosurgical intervention can be performed without delay, increased complications, or involved personnel infection if defined protocols are applied. This record is very important as young children are still not vaccinated and new variant strains of COVID-19 have become predominant.

### Perioperative complications and care

Saynhalath et al. stated that pediatric patients with non-severe COVID-19 infections are at higher risk of developing perianesthetic respiratory complications such as laryngospasm, bronchospasm, hypoxemia, or postoperative supplemental oxygen requirements compared with uninfected patients (9). This is particularly true in patients with previous pulmonary conditions (e.g., bronchopulmonary dysplasia or respiratory distress in premature babies). However, infected children did not show increased risk of non-respiratory complications or mortality and their perianesthetic complication rates are lower compared to adults. These findings were consistent with other reports in literature which also suggested measures to minimize complications and diffusion of the virus (10). For example, the use of intravenous drugs for induction of anesthesia has been demonstrated to lower the rates of perioperative respiratory adverse events compared to inhalation induction.

### Hospital organization and telemedicine

Screening before individuals were allowed to enter the hospital facility was implemented for patients, parents, and healthcare personnel. The COVID-19 pandemic also impacted on other aspects of neurosurgical practice, such as outpatient clinics (3). In order to minimize traffic of patients and family members into the facility, and face-to-face interaction, institutions focused on the improvement and expansion of telemedicine. Both telephone and video-based visits were conducted with success, limiting in-person appointments to those deemed as urgent and absolutely necessary. Optimization of this process allowed full clinic schedules to be maintained even in a situation of restricted in-person contact. Neuroradiological studies were limited to those deemed really necessary.

### Online meetings, education, and working

The same principle of employing electronical technologies was applied to the daily morning report, education, and subspecialty conferences and journal clubs (3). Meetings and handovers were conducted by telephone or video conference. Moreover, in most hospitals, administrative and research staff mainly worked from home. With the reduction of surgical and clinical activities, even medical staff was reduced to an essential core (3). This also helped to always have someone available in case of infection of healthcare personnel. Resident pediatric neurosurgery rotations and students' internships were limited and they were encouraged to focus on research activities (3).

**Figure 1:**
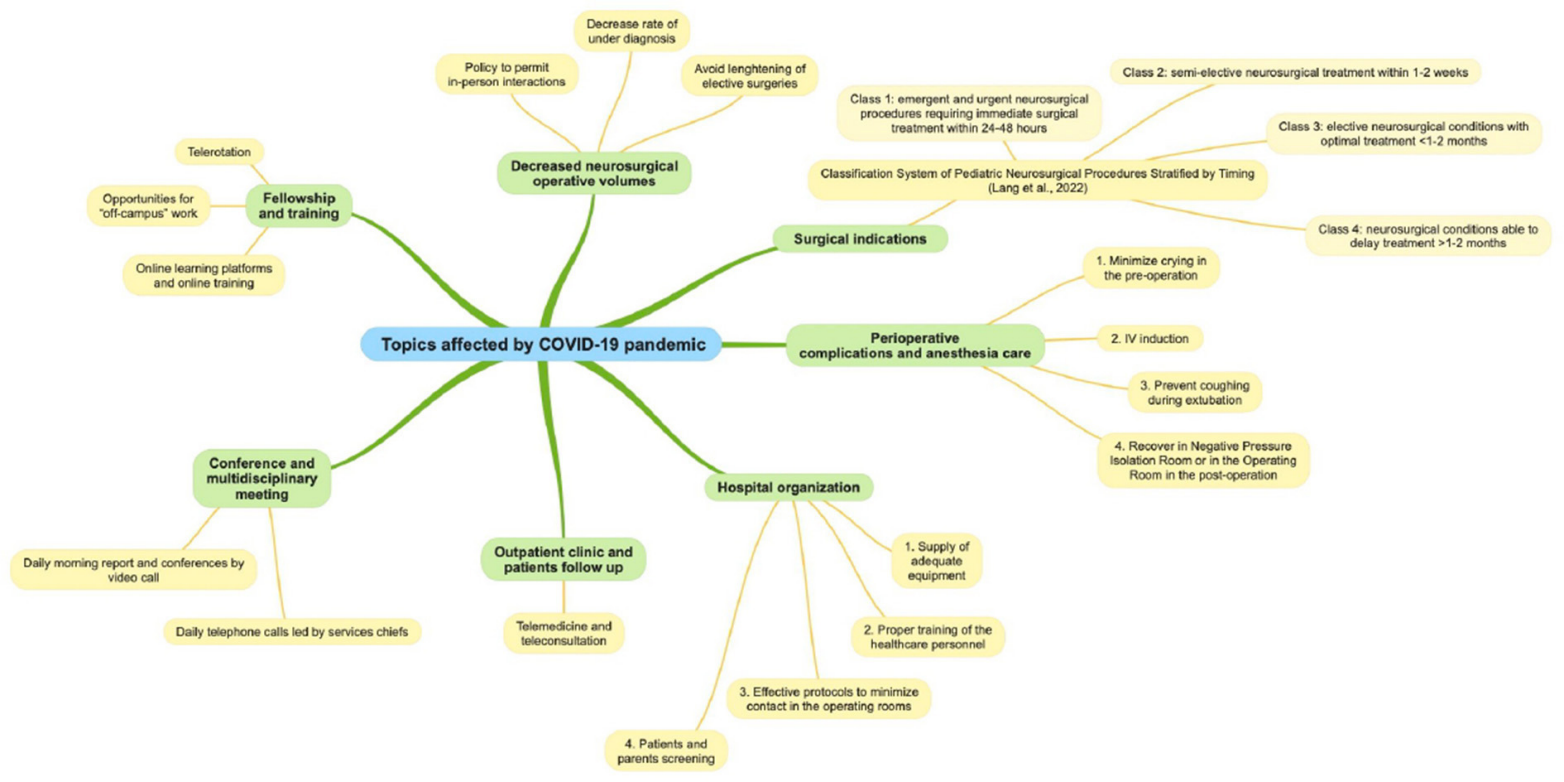
Summary of the most important topics affected by COVID-19 pandemic and related solutions.

A summary of the most important topics affected by the COVID-19 pandemic are presented in Table 1 and Figure 1.

### Meyer children hospital's experience and comparisons

Most of the previously discussed topics give a realistic idea of the measures and strategies undertaken at the Meyer Children's Hospital in Florence during the Covid-19 pandemic. Many similarities were also found comparing the specific guidelines adopted by our hospital and the Giannina Gaslini Children's Hospital in Genoa (11).

One of the main priorities was related to the classification of surgical indications and criteria. This necessity, shared by the two Italian hospitals as well as by the American ones (8), has been approached with a communal principle to stratify the neurosurgical procedures by timing, for a total of four classes (8) and five classes (11).

Concerning the perioperative complications and the anesthetic challenges, the principal precautions taken at our hospital were related to the reduction of aerosol and bag mask ventilation in favor of intravenous drugs administration, and the development of strategies to minimize the virus spread with patient's crying (pre-operation) and coughing (post-operation), in line with the indications proposed by Gai et al. (10).

The same restrictions concerning visitors, patient's assistance, and personnel in the operating room described above were applied in our hospital. For patients' and parents' admission, a nasopharyngeal swab must be performed within 48 h.

Both personnel and visitors had to be monitored with routine screening and wear adequate PPE, as comprehensively studied by Ballestero et al. (1).

Another very challenging aspect that had to be faced was related to the education of residents and fellows and, as described by Weiner et al., the development of online opportunities and “off-campus” solutions represented the key factor (3). At Meyer Children's Hospital, face-to-face lectures and multidisciplinary reunions were substituted with online meetings and video conferences. We also had direct proof that telemedicine represents a valid alternative for outpatient clinics and patient follow up, in order to reduce face-to-face interactions and high numbers of visitors to the hospital.

In conclusion, we are prone to state a remarkable homogeneity in the guidelines adopted by hospitals in Europe, South America, Canada, and the USA.

## Conclusion

The above-described topics are only part of the profound change produced by the COVID-19 pandemic to pediatric neurosurgery.

The aim of this study was firstly to evaluate the level of evidence and new guidelines proposed by up-to-date literature. In the second instance, we compared our hospital's experience with the strategies undertaken to face COVID-19 by different healthcare systems all over the world, in order to offer an innovative point of view on the current pediatric neurosurgical trends.

Although epidemiological data look more promising now and the availability of the vaccine even for the youngest children is helping us deal with this challenging battle against the virus, the advent of new very contagious variants continuously creates new obstacles. For these reasons, we believe that further studies and research will be necessary.

## Data availability statement

The original contributions presented in the study are included in the article/supplementary material, further inquiries can be directed to the corresponding author/s.

## Author contributions

FG conceived the study. AN, SP, and CS collected and re-examined the data and wrote the manuscript. FG, AD, and ML reviewed and revised the manuscript. All authors contributed to the article and approved the submitted version.

## Funding

This article has been supported and founded by Meyer Children's Hospital Foundation, Florence.

## Conflict of interest

The authors declare that the research was conducted in the absence of any commercial or financial relationships that could be construed as a potential conflict of interest.

## Publisher's note

All claims expressed in this article are solely those of the authors and do not necessarily represent those of their affiliated organizations, or those of the publisher, the editors and the reviewers. Any product that may be evaluated in this article, or claim that may be made by its manufacturer, is not guaranteed or endorsed by the publisher.
